# Reliability, validity, sensitivity and internal consistency of the ICF based Basic Mobility Scale for measuring the mobility of patients with musculoskeletal problems in the acute hospital setting: a prospective study

**DOI:** 10.1186/s12891-015-0638-7

**Published:** 2015-08-05

**Authors:** Karin Pieber, Malvina Herceg, Tatjana Paternostro-Sluga, Eleonore Pablik, Michael Quittan, Peter Nicolakis, Veronika Fialka-Moser, Richard Crevenna

**Affiliations:** Department of Physical Medicine and Rehabilitation, Medical University of Vienna General Hospital of Vienna, Waehringer Guertel 18-20, A-1090 Vienna, Austria; Section for Medical Statistics, Center for Medical Statistics, Informatics and Intelligent Systems, Medical University of Vienna, Vienna, Austria; Institute of Physical Medicine and Rehabilitation and Karl Landsteiner Institute of Remobilization and Functional Health, Kaiser Franz-Joseph Hospital, Vienna, Austria

**Keywords:** Assessment of mobility, Psychometric properties, FIM, Ageing population

## Abstract

**Background:**

The assessment of mobility is important in the acute care setting. Existing tests suffer from limitations. The aim of the study was to examine the inter-rater reliability, the validity, the sensitivity to change, and the internal consistency of an ICF based scale.

**Methods:**

In a prospective study inpatients in the acute care setting with restricted mobility aged above 50 years assigned to rehabilitative treatment were included. Assessment of subscales of the Functional Independence Measure (FIM) and the ICF based Basic Mobility Scale (BMS) were performed at admission and before discharge. Furthermore pain, length of stay in hospital, and post-discharge residential status were recorded. Inter-rater reliability, criterion-concurrent validity, sensitivity to change, and internal consistency were calculated. Furthermore, floor and ceiling effects were determined.

**Results:**

One hundred twenty-five patients (79 women/46 men) were included. The BMS showed an excellent inter-rater reliability for the total BMS (ICC BMS: 0.85 (95 % CI: 0.81–0.88). The criterion-concurrent validity was high to excellent (Spearman correlation coefficient: −0.91 in correlation to FIM) and the internal consistency was good (Cronbach’s alpha 0.88). The BMS proved to be sensitive to improvements in mobility (Wilcoxon’s signed rank test: *p* < 0.0001; The effect size for the BMS was 1.075 and the standardized response mean 1.10. At admission, the BMS was less vulnerable to floor effects.

**Conclusions:**

The BMS may be used as a reliable and valid tool for the assessment of mobility in the acute care setting. It is easy to apply, sensitive to change during the hospital stay and not vulnerable to floor and ceiling effects.

**Electronic supplementary material:**

The online version of this article (doi:10.1186/s12891-015-0638-7) contains supplementary material, which is available to authorized users.

## Background

The functional decline of patients has been reported as a result of hospitalization and is pronounced in the older population [1]. Early mobilization of patients in the acute care setting is of utmost importance to decrease length of stay and avoid permanent impairments [2]. Diminished independence is associated with an increased risk of transfer to nursing home, caregiver burden [3], mortality and healthcare costs after discharge especially of older patients [4]. One of the most important aspects of functional decline during hospitalization is reduced mobility which may have an impact on independence and quality of life. In 2009, the mobility of 500 Inpatients (aged between 20 and 99 years) was evaluated in a multicenter study in different hospitals in Vienna. A highly significant correlation was found between mobility and length of stay in hospital and mortality rate. Patients with impaired mobility stayed in the hospital longer and presented a higher mortality rate [5].

Floor and ceiling effects are the major problems of existing tools for assessing mobility in the acute care setting. These limitations have been reported for the Timed Up and Go test (TUG) [6], the Functional Reach Test (FRT) [7], and activities of daily living scales such as the Barthel Index (BI) [8] and Functional Independence Measure (FIM) [9, 10]. Cohen and Marino reported on floor and ceiling effects of the BI in post stroke patients and patients with recent hip fractures, as well as substantial ceiling effects of the FIM cognition items in patients at rehabilitation discharge with spinal cord injuries [11]. Stineman *et al.* detected only small floor and ceiling effects for the FIM for most items and most patients suffering from 20 diverse impairment categories [12]. Other scales like the de Morton Mobility Index (DEMMI) [13] or the Functional Status Score for the Intensive Care Unit (FSS-ICU) [14] are valid and reliable tools for assessing mobility but only in a specific group of patients (older than 65 years) or in a specific setting (geriatric setting, not acute care setting, ICU). Furthermore, most scales require a certain amount of mobility, e.g. walking.

In a health care system, aiming at shorter hospital stay despite shortening in personal resources, a more detailed scale is required to adequately target specific interventions. This scale has to be capable to assess the functional ability from his first day in hospital, requiring a fine tuned scale, assessing the patient from completely bed-ridden (e.g. intensive care unit) to independent walking, including the activities necessary to independently get out of bed.

The International Classification of Functioning, Disability and Health (ICF) is a worldwide accepted concept of functional health, providing, among others, categories for all aspects of body functions, activities and participation [15]. Furthermore the basics of ICF are well known in literature and daily routines and therefore the scale can be easily explained and applied. Following this concept, the ICF provides a range of validated core sets for different conditions [2, 16, 17]. Additionally, quantification of categories is at its beginning thus providing no valid measurements.

Up to now, no generic core sets exist for very early mobilization of bed-ridden and critically ill patients. Nevertheless, out of existing core sets [16], categories can be extracted fitting the aim of a newly developed scale. A new generic scale, termed Basic Mobility Scale (BMS) should cover the entire range of mobility from bed-ridden to independent mobility for a wide range of clinical patterns. It should be feasible in daily routines, sensitive to changes during the hospital stay and psychometrically robust. Therefore, the objective of this study was to examine the reliability, the validity, the sensitivity to change, and the internal consistency of the Basic Mobility Scale.

## Methods

The study was performed between October 2012 and January 2013 according to the declaration of Helsinki and was approved by the Ethical Review Board of the Medical University of Vienna (EK No. 337/2010, chairman: Prof. Singer). Informed consent was obtained from all participants.

### Study design, setting and patients

This prospective study was conducted at the General Hospital of Vienna, which is 2112 bed facility (in 2014). All eligible Inpatients with restrictions in mobility due to musculoskeletal problems or deconditioning from the Department of Orthopedics, Trauma-Surgery and Internal Medicine were consecutively included over a period of 4 months. Inclusion criteria were defined as the following factors: age above 50 years, patients with impaired mobility due to surgery or an accident or deconditioning affecting the musculoskeletal system as reason for admission, rehabilitative treatment during their stay, understanding of German and written informed consent. We excluded patients with cancer or psychiatric illness.

### Description and development of the BMS

We intended to create a scale, which covers the mobility of the patient from bed-ridden to stair climbing. Suitable items out of existing tools were detected by experts’ opinions, and connected with appropriate ICF items or sub-items in the appropriate ICF-Core-Sets. So, the BMS has been generated to an instrument for evaluating the mobility of patients based on the ICF-Core-Sets in Acute Settings and Early Rehabilitation for Patients with Musculo-Skeletal Conditions [2, 16, 18]. The scale consists of six items, which reflect the most important activities of patients in the acute care setting and is focused on more restricted mobility levels. The items are labelled BMS 1 “Changing position while lying” (correspondent to d410 Changing basic body position), BMS 2 “Maintaining a sitting position” (sitting on the edge of bed, correspondent to d415 Maintaining a body position), BMS 3 “Maintaining a standing position” (correspondent to d415 Maintaining a body position), BMS 4 “Transferring oneself” (correspondent to d420 Transferring oneself), BMS 5 “Walking short distances” (correspondent to d450 Walking) and BMS 6 “Climbing stairs” (correspondent to d4551 Climbing stairs). The items are not scored hierarchically. To describe the mobility of the patient more in detail, we defined sub-items. These sub-items contain a quantitative scoring like a grading concerning the achieved duration or distance and defines e.g. how long a patient can sit, how many meters the patient can walk or how many steps the patient can take. Each item and/or sub-item has a scoring concerning the quality from a: total independence; b: with crutches/stick (e.g. after total knee or hip arthroplasty for a predetermined period); c: with aids like walking frame; d: with the help of one person; e: with the help of two persons to f: not possible. Not applicable portions were shaded. The scoring refers to the active skills of the patients and can be evaluated during rehabilitative treatment. Each of the six items has to be tested and one box has to be ticked off. Behind each box there is a score (scoring matrix). The total score is the addition of the scores of all six items. This provides a total possible score of 6 (which represents independent mobility) to the maximum score of 70 (completely dependent) (see Additional file 1: Basic Mobility Scale). The qualitative and quantitative scoring of these items was created and refined to evaluate the patient’s mobility as well as the possible need for additional devices in hospital. The BMS was tested in daily routines and adapted to the requirements of the performing health professionals. The scoring matrix was developed by two experienced specialists in Physical Medicine and Rehabilitation and two experienced physiotherapists.

### Data collection procedures

The recruitment, informed consent and randomization concerning the rater sequence were performed by two doctors, both which are specialists in the field of rehabilitation. All outcome measures were assessed at admission and before discharge by four health professionals (two teams consisting of two raters). Before starting the study all contributing health professionals discussed the used assessment tools to provide clarity and guidance on how to rate each item.

### Measures

#### Assessment of inter-rater reliability

To evaluate the inter-rater reliability patients were examined alternating by two independent raters. One rater conducted the assessments in the morning, the other one in the afternoon. Between the evaluations, a resting time of at least 2 hours was provided. The sequence was randomly assigned by a randomization protocol of the Institute for Medical Statistics of the Medical University of Vienna. All raters used the same protocol.

#### Assessment of criterion-concurrent validity

Criterion-concurrent validity was evaluated by means of the correlation between the BMS and subscales of the FIM, which include transfers and locomotion (transfers: bed/chair/wheelchair, toilet and bath tub/shower; locomotion: walking or wheelchair and climbing stairs). The scores range from 1 (total assistance) to 7 (total independence). For the Total FIM score we added the scores of all subscales (range 3–21). We intended to measure how well the results of the BMS relate to data obtained from a gold standard instrument - the FIM subscales. We used the FIM subscales as they are most similar to our scale and are broadly used in hospitals in Germany and Austria. The motor FIM was shown to be valid, reliable and able to detect changes in disability [19] and was successfully used in a recent study dealing with patients after hip fracture [20]. Previously, some studies used only parts of the FIM in community dwelling elderly people [21], for use in long-term care setting [22], or spinal cord injuries [23] and described their score as valid [22], sensitive and specific [21], and reliable [23]. Our physiotherapists had been well trained in evaluating mobility using the FIM in advance.

#### Assessment of sensitivity to change

To analyze the sensitivity to improvements in mobility during the hospital stay (rehabilitative treatment being performed during the stay) we evaluated the patients at their admission and discharge.

For better description of our patients, we evaluated pain and the subjective rating of mobility by using the visual analogue scale (VAS; a 10 point scale with ends labeled from no pain/no limitation to worst possible pain/worst possible limitation), length of stay in hospital (in days) and post-discharge residential status (home, nursing home for the elderly, Acute Geriatrics and Remobilization facility, Acute Geriatric Unit or another hospital/another ward in the same hospital). Furthermore, we interviewed our four raters after the study if they would use the BMS in their daily routines within the rehabilitative treatment. Moreover, floor and ceiling effects of the BMS, the FIM subscales and the Total FIM score were determined using the percentage of occasions when patients scored the lowest or highest possible score for the scale. This method was already used by Parry *et al*. [24].

### Statistical analysis

Data were entered into Microsoft Excel and analyzed using the Statistical Package for Social Sciences (SPSS) Version 15.0 and R (Version 2.15.2).

#### Assessment of inter-rater reliability

To assess the inter-rater reliability of the BMS, the agreement between the two independent raters was calculated with the intra-class correlation coefficient (ICC (1,1) or “one-way” ICC).

#### Assessment of criterion-concurrent validity

The correlation of the BMS and the Total FIM score was examined by Spearman correlation coefficients for the first and second evaluation (same patient, same evaluation time, same rater).

#### Assessment of sensitivity to change

Wilcoxon’s signed rank test was used to determine the sensitivity of the BMS to improvements in mobility of the patients during their stay. Furthermore, for the responsiveness to change of the scale the effect size (ES) and the standardized response mean (SRM) will be presented.

#### Assessment of internal consistency

As the BMS is the sum of six different items, we analyzed the internal consistency with Cronbach’s alpha and the Pearson correlation coefficients of the items and the total score.

## Results

### Patients’ characteristics, data from admission and discharge

We included 125 patients (79 women and 46 men) in this study. Complete data at admission and discharge was available from 105 patients. Lost data were due to unexpected early discharge (*n* = 19) and death (*n* = 1). Most patients were allocated from the Department of Orthopedics and most of the restrictions in mobility were due to operations of the hip, knee and spine. Five patients from the Department of Internal Medicine suffered from restrictions in mobility due to deconditioning. At admission, patients rated their mobility as moderately restricted and the BMS and Total FIM score provided a moderate restriction in mobility (32 out of 70 points and 10 out of 21 points). Patients’ characteristics, allocating Department, diagnosis, pain, subjective rating of mobility, Basic Mobility Scale and Total FIM score at admission are presented in Table 1 in detail. At discharge, patients rated their restrictions in mobility less and the BMS and Total FIM score reflected only light restrictions in mobility (16 out of 70 points and 17 out of 21 points). Number of received rehabilitative treatment during hospital stay, length of stay, pain, subjective rating of mobility, Basic Mobility Scale and Total FIM score at discharge as well as post-discharge residential status are shown in Table 2 in detail.

**Table 1:** Patients’ characteristics, allocating Department, diagnosis, pain, subjective rating of mobility, Basic Mobility Scale at admission and Total Functional Independence Measure at admission

	Mean (SD)/Amount
Age (years)	
Mean	67.2 (9.2)
Min-max	50–90
Height (cm)	
Mean	168.7 (8.9)
Min-max	149–200
Weight (kg)	
Mean	79.7 (17.0)
Min-max	46–127
From the Department of	
Orthopedics	96
Trauma-Surgery	23
Internal Medicine	5
Diagnosis	
Total knee arthroplasty	52
Total hip arthroplasty	32
Proximal femur nail	8
Spinal surgery	7
Others (e.g. deconditioning due to chronic respiratory disease, lung transplantation, or chronic renal failure)	26
Pain at admission (VAS)	
Mean	3.7 (2.7)
Min-max	0–10
Subjective rating of mobility (VAS)	
Mean	5.1 (2.8)
Min-max	0–10
BMS at admission (*n* = 125)	
Mean	32.30 (14.95)
Min-max	8–68
Total FIM score at admission (*n* = 125)	
Mean	10.08 (4.07)
Min-max	3–21

*BMS*, basic mobility scale, *VAS* visual analogue scale (0: no pain/no limitations—10: worst possible pain/worst possible limitations), *Total FIM score* addition of all FIM subscales (Functional Independence Measure)

**Table 2:** Number of received rehabilitative treatment during hospital stay, length of stay, pain, subjective rating of mobility, Basic Mobility Scale at discharge, Total Functional Independence Measure at discharge and post-discharge residential status

	Mean (SD)/Amount
Received rehabilitative treatment (sessions)	
Mean	6.4 (3.8)
Min-max	1–34
Length of stay (days)	
Mean	13.3 (7.6)
Min-max	4–76
Pain at discharge (VAS)	
Mean	2.6 (2.4)
Min-max	0–9
Subjective rating of mobility at discharge (VAS)	
Mean	3.6 (2.6)
Min-max	0–10
BMS at discharge (*n* = 105)	
Mean	15.68 (7.93)
Min-max	6–47
Total FIM score at discharge (*n* = 105)	
Mean	16.69 (3.27)
Min-max	3–20
Discharge to	
Home	94
Back to nursing home for the elderly	1
An acute geriatrics and remobilization facility	13
An acute geriatric unit	7
Another hospital/another ward in the same hospital	9

*BMS* basic mobility scale, *VAS* visual analogue scale (0: no pain/no limitations—10: worst possible pain/worst possible limitations), *Total FIM score* addition of all FIM subscales (Functional Independence Measure)

Pain and subjective rating of mobility significantly improved during hospital stay (*p* = 0.006 and *p* = 0.0001). All four raters deemed the BMS feasible for daily routine, since the administration of the BMS did not interfere with the applied therapy as all items are routinely assessed during the rehabilitative treatment. Floor and ceiling effects are presented in Table 3.

**Table 3:** Floor and ceiling effects of the Basic Mobility Scale and the FIM subscales presented in percentage of occasions when patients scored the lowest or highest score possible for the scale

	Admission		Discharge	
	Floor	Ceiling	Floor	Ceiling
BMS	0	0	0.9 %	0
FIM climbing stairs	96 %	0.8 %	19 %	2 %
FIM transfer	10 %	7 %	0.9 %	24 %
FIM walking	29 %	0.8 %	3 %	4 %
Total FIM score	9 %	0.8 %	0.9 %	0

#### Inter-rater reliability

The BMS showed an excellent inter-rater reliability in the total BMS and moderate to very good inter-rater reliability in the items (ICC BMS: 0.85 (95%CI: 0.81–0.88, ICC items: BMS 1: 0.61 (0.53–0.69), BMS 2: 0.78 (0.72–0.82), BMS 3: 0.72 (0.66–0.78), BMS 4: 0.73 (0.66–0.78), BMS 5: 0.78 (0.72–0.82), BMS 6: 0.89 (0.87–0.92)).

#### Criterion-concurrent validity

**Fig. 1:**
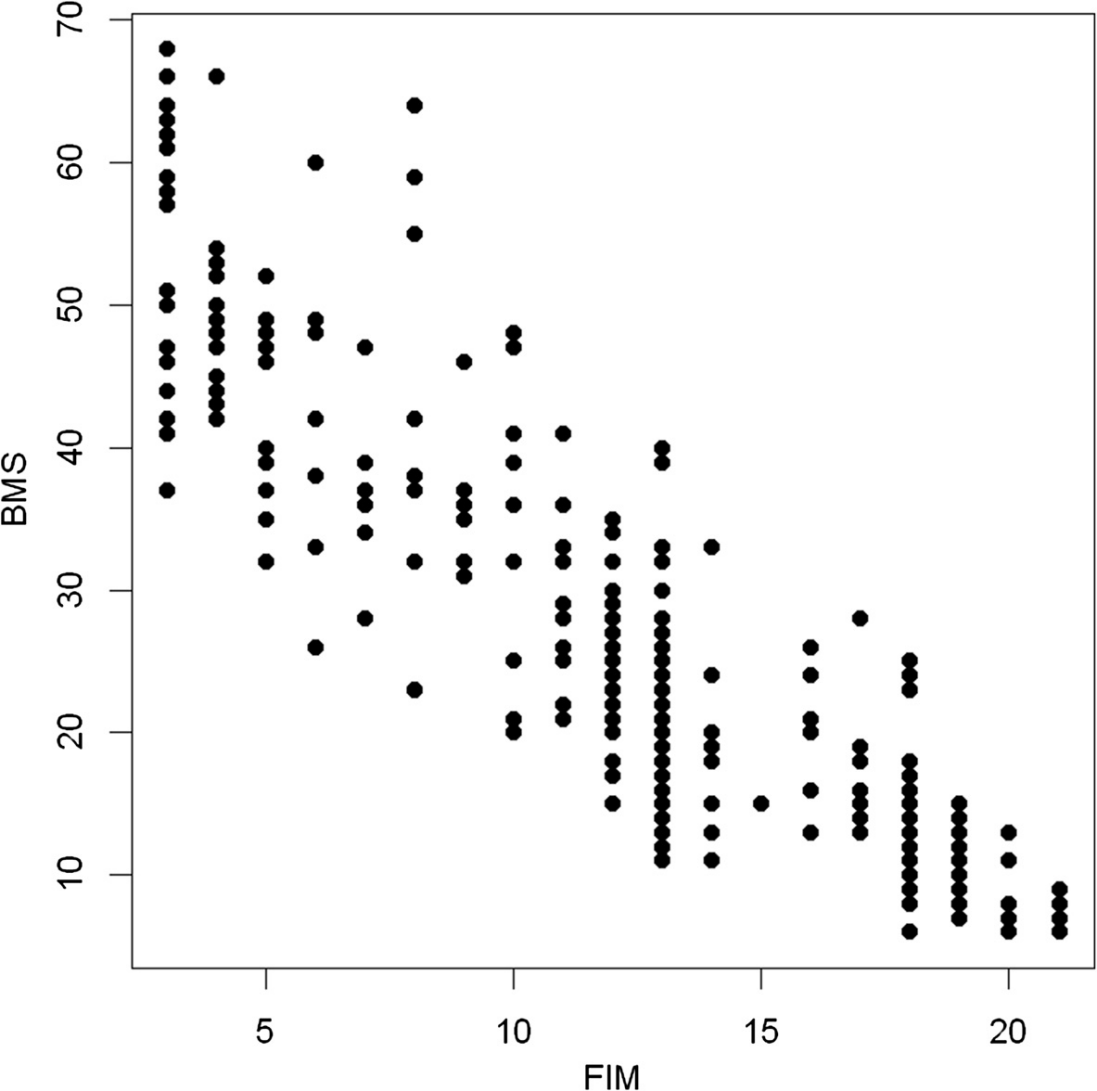
Correlation of the Basic Mobility Scale (BMS) with the sum of the subscales of the Functional Independence Measure (FIM)

Total FIM score and BMS were significantly negatively correlated (Spearman correlation coefficient: −0.91). For more detail see Fig. 1. This correlation was slightly stronger at admission than at discharge, which might be due to the very low variance between the patients at discharge (Spearman BMS-FIM at admission: −0.86 (*p* < 0.0001) and at discharge: −0.78 (*p* < 0.0001)).

#### Sensitivity to change

The BMS decreased about the half from admission to discharge which represented an improvement in mobility. The BMS proved to be sensitive to improvements in mobility (Wilcoxon’s signed rank test: *p* < 0.0001, ES for the BMS 1.075 and SRM 1.10).

#### Internal consistency

Cronbach’s alpha of the total BMS was 0.875. All correlations between the items and the total score were clearly above 0.2 with no substantial elevation of Cronbach’s alpha in case of elimination of one item. For details see Table 4.

**Table 4:** Internal consistency of the Basic Mobility Scale presented with Cronbach’s alpha and the Pearson correlation coefficients of the items and the total score

		Cronbach’s alpha
BMS		0.875
	Pearson correlation of the items with total score	Cronbach’s alpha if item is deleted
BMS 1	0.583	0.8698
BMS 2	0.667	0.8551
BMS 3	0.831	0.8387
BMS 4	0.828	0.8367
BMS 5	0.834	0.8295
BMS 6	0.532	0.8827

BMS 1 “Changing position while lying”, BMS 2 “Maintaining a sitting position” (sitting on the edge of bed), BMS 3 “Maintaining a standing position”, BMS 4 “Transferring oneself”, BMS 5 “Walking short distances” and BMS 6 “Climbing stairs”

## Discussion

According to our data, the BMS maybe a reliable, valid and sensitive tool for evaluation of mobility in patients with restricted mobility due to musculoskeletal problems in the acute care setting.

Most of the already existing scales for the assessment of mobility suffer from limitations concerning floor and ceiling effects, missing information concerning the responsiveness to change and were mostly created for a specific population (stroke, geriatric) or specific setting (outpatient, rehabilitation) [25–29]. Furthermore, these scales and tests do not provide detailed information about the mobility of the patient from bed-ridden to stair climbing.

For the TUG test, a floor effect is described with approximately one quarter of hospitalized older people unable to complete it because they are too weak [25]. The BI has a ceiling effect when used with patients who are independently ambulant [26]. Furthermore, the reported ceiling effect of the Elderly Mobility Scale (EMS) at discharge of the hospital restricts the validity for assessing mobility of older patients in the acute care setting [27]. We could not detect floor or ceiling effects for the BMS at admission, in contrast to the FIM climbing stairs, the FIM transfer, the FIM walking and the Total FIM score. At discharge, the BMS and the Total FIM score presented no floor or ceiling effect but a floor effect could be found for the FIM climbing stairs and a ceiling effect for the FIM transfer.

The new scale demonstrated a good internal consistency comparable to values of other mobility tools [12, 30]. The elimination of one item did not improve the internal consistency substantially. This indicates that every item contributes well to the total scale and no individual item detracted from the integrity of the BMS as a whole. The inclusion of items which are simple (like BMS 1 “Changing position while lying”) but also items which are difficult to perform (like BMS 6 “Climbing stairs”) could have contributed to overcome any potential ceiling and floor effects. Additionally, each item of the BMS includes a wider range of levels (Fig. 1) which makes the scale more sensitive.

Some instruments have an inadequate scale width to detect changes in mobility for people whose limitations are either severe or relatively modest [26]. The two Minute Walk Test [31], EMS [32] or Rivermead Mobility Index [33] are mostly used for more mobile patients and for the assessment of functional mobility following stroke or other neurological diseases. Functional independence or activities of daily living can be further assessed by the use of the Modified Rankin scale [28] or the Frenchay Activities Index [29], both mostly used for patients after stroke and therefore a specific population. More extensive and time consuming tests for the evaluation of mobility and activities are the 22-item Mobility Activities Measure [34] and the AM-PAC Activity Domains [35], which are used in the outpatient rehabilitation setting and not in the acute care in hospital. The new generic scale can be used for all patients with restricted mobility due to musculoskeletal problems in the acute care setting and describes the mobility from bed-ridden to independently mobile patients. Criterion-concurrent validity between BMS and the Total FIM score was high to excellent which shows that the BMS was a valid assessment tool for mobility in this sample. The high correlation may be explained by the similar items of each scale. The Spearman correlation coefficient was comparable to the correlation of EMS with BI (Spearman correlation coefficient 0.96) and FIM (Spearman correlation coefficient 0.95) in Inpatients aged 70–93 years [26] and better than the correlation of BI with the Hierarchical Assessment of Balance and Mobility (Spearman correlation coefficient 0.76) in older acute medical patient population [26].

The BMS revealed an excellent inter-rater reliability comparable to a study by Hamilton *et al.* [9]. The inter-rater reliability for the subscales transfers and locomotion of the FIM were between 0.57 and 0.66.

The sensitivity to change of the scale was confirmed by the possibility to present statistically significant differences in mobility during the hospital stay and a very high ES. This is a very important characteristic of a tool since it makes it possible to detect improvements or worsening in mobility and furthermore to determine the therapeutic effectiveness when performing rehabilitative treatment during the stay. Therefore, the BMS may be used in quality management as benchmarking tool for Inpatients.

For a safe discharge a sufficient level of mobility is important and the BMS may assist with discharge planning as it reflects the mobility and the required amount of assistance and is easily applicable in daily routines in the acute care setting. Certainly, much more factors need to be considered to decide if there is a possibility to be discharged to home or whether there is the need of assistance in other facilities.

### Study limitations

Most patients were from the Department of Orthopedics undergoing hip or knee arthroplasty with temporary mobility deficits mostly due to postoperative standards. For inter-rater reliability, only two raters assessed the patients. This applied methodology was already used in a study by De Morton in 2010 [13] dealing with the validity and reliability of the DEMMI in a geriatric rehabilitation setting and in a study by Stubbs in 2014 [36] about the FIM in patients with acquired brain injury. Furthermore, we did not assess the administration time but all four raters reported that the BMS was easy to apply and that they would be able to incorporate it in their daily routines. If the statistically significant changes are also clinically relevant was not evaluated. It is not possible to generalize our results for patients different to our patients with restricted mobility due to musculoskeletal problems in the acute hospital setting. Further investigations on the use of the BMS evaluating the mobility of other patients’ samples with, e.g., more severe impairments in mobility, like at the ICU or geriatric patients are planned.

## Conclusions

The BMS might be a valid and reliable tool for the assessment of mobility in the acute care setting which is sensitive to even smaller changes and provides detailed information concerning the mobility of the patient from bed-ridden to stair climbing. It is easily applicable in daily routines during the rehabilitative treatment of patients with musculoskeletal problems and has the major advantage not suffering from floor or ceiling effects.

## Additional file

Additional file 1:
**Basic mobility scale.** (DOCX 20 kb)

## Abbreviations

TUG: Timed up and go test
FRT: Functional reach test
BI: Barthel index
FIM: Functional independence measure
DEMMI: De Morton mobility index
BMS: Basic mobility score
EMS: Elderly mobility scale
ICF: International Classification of Functioning
VAS: Visual analogue scale
ICC: Intra-class correlation coefficient
ES: Effect size
SRM: Standardized response mean
ICU: Intensive care unit
