# Supplementary material for: Reliability, validity, sensitivity and internal consistency of the ICF based Basic Mobility Scale for measuring the mobility of patients with musculoskeletal problems in the acute hospital setting: a prospective study
Source: BMC Musculoskelet Disord. 2015 Aug 5;16:187. doi: 10.1186/s12891-015-0638-7 (PMC4525726; doi:10.1186/s12891-015-0638-7)
Supplement: Additional file 1: — Basic mobility scale. (DOCX 20 kb) [file 12891_2015_638_MOESM1_ESM.docx]

**Basic Mobility Scale** Version 2.0, 09.02.2015

**1 Changing position while lying**

| **a** | **b** | **c** | **d** | **e** | **f** |
| --- | --- | --- | --- | --- | --- |
| **1** |  | **4** | **6** | **8** | **10** |

**2 Maintaining a sitting position (Sitting on edge of bed - EOB)**

|  | **a** | **b** | **c** | **d** | **e** | **f** |
| --- | --- | --- | --- | --- | --- | --- |
| < 1 min | **9** |  | **10** | **13** | **14** | **15** |
| 1 - 14 min | **3** |  | **6** | **10** | **12** |  |
| 15 min – 59 min | **2** |  | **5** | **8** | **11** |  |
| ≥ 1 h | **1** |  | **4** |  |  |  |

**3 Maintaining a standing position**

|  | **a** | **b** | **c** | **d** | **e** | **f** |
| --- | --- | --- | --- | --- | --- | --- |
| < 1 min | **3** | **4** | **5** | **8** | **9** | **10** |
| 1 - 4 min | **2** | **3** | **4** | **7** | **8** |  |
| ≥ 5 min | **1** | **2** | **3** | **6** | **7** |  |

**4 Transferring oneself** (armchair/wheelchair)

| **a** | **b** | **c** | **d** | **e** | **f** |
| --- | --- | --- | --- | --- | --- |
| **1** | **2** | **4** | **6** | **8** | **10** |

**5 Walking short distances**

|  | **a** | **b** | **c** | **d** | **e** | **f** |
| --- | --- | --- | --- | --- | --- | --- |
| < 5 m | **5** | **6** | **7** | **10** | **13** | **15** |
| 5 – 9 m | **4** | **5** | **6** | **9** | **12** |  |
| 10 – 19 m | **3** | **4** | **5** | **8** | **11** |  |
| 20 – 49 m | **2** | **3** | **4** | **7** | **10** |  |
| ≥ 50 m | **1** | **2** | **3** | **6** | **9** |  |

**6 Climbing stairs** (b = with handrail, c = with handrail and devices)

|  | **a** | **b** | **c** | **d** | **e** | **f** |
| --- | --- | --- | --- | --- | --- | --- |
| < 6 steps | **4** | **5** | **6** | **8** | **9** | **10** |
| 6 - 12 steps | **3** | **4** | **5** | **7** | **8** |  |
| 13 - 23 steps | **2** | **3** | **4** | **6** | **7** |  |
| ≥ 24 steps | **1** | **2** | **3** | **5** |  |  |

**a** = independent, **b** = with crutches/walking stick (e.g. after total hip arthroplasty for a predetermined period), **c** = with device (e.g. walking frame; EOB sitting device), **d** = with 1 person**, e** = with 2 persons, **f** = not possible, shaded portions = not applicable.

Evaluate each item and sub-item concerning the ability to perform it independently or with different types of assistance as well concerning the achieved duration or distance of the tested item. The scoring refers to the active skills of the patients and can be evaluated during rehabilitative treatment.
